# Tracking SARS-CoV-2 Omicron diverse spike gene mutations identifies multiple inter-variant recombination events

**DOI:** 10.1038/s41392-022-00992-2

**Published:** 2022-04-26

**Authors:** Junxian Ou, Wendong Lan, Xiaowei Wu, Tie Zhao, Biyan Duan, Peipei Yang, Yi Ren, Lulu Quan, Wei Zhao, Donald Seto, James Chodosh, Zhen Luo, Jianguo Wu, Qiwei Zhang

**Affiliations:** 1 Guangdong Provincial Key Laboratory of Virology, Institute of Medical Microbiology, Jinan University, 510632 Guangzhou, China; 2 BSL-3 Laboratory (Guangdong), Guangdong Provincial Key Laboratory of Tropical Disease Research, School of Public Health, Southern Medical University, 510515 Guangzhou, China; 3 Bioinformatics and Computational Biology Program, School of Systems Biology, George Mason University, Manassas, VA 20110 USA; 4 Department of Ophthalmology, Howe Laboratory Massachusetts Eye and Ear, Harvard Medical School, Boston, MA 02114 USA; 5 Foshan Institute of Medical Microbiology, 528315 Foshan, China

**Keywords:** Microbiology, Infectious diseases

## Abstract

The current pandemic of COVID-19 is fueled by more infectious emergent Omicron variants. Ongoing concerns of emergent variants include possible recombinants, as genome recombination is an important evolutionary mechanism for the emergence and re-emergence of human viral pathogens. In this study, we identified diverse recombination events between two Omicron major subvariants (BA.1 and BA.2) and other variants of concern (VOCs) and variants of interest (VOIs), suggesting that co-infection and subsequent genome recombination play important roles in the ongoing evolution of SARS-CoV-2. Through scanning high-quality completed Omicron spike gene sequences, 18 core mutations of BA.1 (frequency >99%) and 27 core mutations of BA.2 (nine more than BA.1) were identified, of which 15 are specific to Omicron. BA.1 subvariants share nine common amino acid mutations (three more than BA.2) in the spike protein with most VOCs, suggesting a possible recombination origin of Omicron from these VOCs. There are three more Alpha-related mutations in BA.1 than BA.2, and BA.1 is phylogenetically closer to Alpha than other variants. Revertant mutations are found in some dominant mutations (frequency >95%) in the BA.1. Most notably, multiple characteristic amino acid mutations in the Delta spike protein have been also identified in the “Deltacron”-like Omicron Variants isolated since November 11, 2021 in South Africa, which implies the recombination events occurring between the Omicron and Delta variants. Monitoring the evolving SARS-CoV-2 genomes especially for recombination is critically important for recognition of abrupt changes to viral attributes including its epitopes which may call for vaccine modifications.

## Introduction

The current COVID-19 pandemic is fueled by a more infectious emergent Omicron variant (B.1.1.529), which was first reported in South Africa and quickly spread worldwide.^1^ A multitude of mutations (more than 30) in the spike gene (S) of Omicron variant were detected, which when compared to the Alpha and Delta variants (typically less than 15),^2^ raised concerns of enhanced infectivity and immune escape potential.^3,4^ Omicron variants is divided into three lineages (BA.1, BA.2, and BA.3) and was classified as the fifth variant of concern (VOC) by the World Health Organization on 26 November 2021. It has been circulating in more than 170 countries/territories.

Mutations in the SARS-CoV-2 spike gene have altered protein binding efficiency and immunogenicity, and resulted in more invasive and adaptive variants.^4–9^ Previous research on Alpha (B.1.1.7) and Delta (B.1.617.2 and AY.x) variants with spike gene mutations confirmed these effects on enhancing virus transmission.^6–8,10^ Meanwhile, as a critical antigenic recognition site, the spike protein is also the principal vaccine design target, and these observed mutations have focused attention on this modified antigen and its putative immune escape potential and antibody resistance.^3,11,12^

Ongoing concerns of emergent variants includes possible recombinants resulting from different variants replicating simultaneously in immunocompromised or unvaccinated populations. Such variants, e.g., “Deltacron” or “Demicron” are controversial that if they are real recombinants or a possible sequencing error.^13^

Genome recombination is an important evolutionary mechanism for the emergence and re-emergence of human pathogens and a major source of viral evolution, for example, the well-studied “model organism” adenovirus,^14–20^ and also in coronaviruses.^21–24^ Recombination accelerates virus evolution through gene(s) and “function” transference and accumulation of selective and advantageous mutations, resulting in phenotype changes that include changes in pathogenicity profiles, host species virulence, zoonotic and anthroponotic transmission, and host adaptation.^14,21,22,24,25^

Although recombination events among SARS-CoV and MERS-CoV were well-documented,^21,22,26^ it has been difficult to detect the recombination signatures in SARS-CoV-2 variants due to the high degree of sequence similarity amongst SARS-CoV-2 isolates and the incomplete coverage of coronaviruses from other hosts, including pangolin.^27,28^

Previous research distinguished active recombination events among the SARS-CoV-2 nucleoprotein and ORF1ab genes by using a phylogenetic network strategy based on single nucleotide substitution or SARS-CoV-2 lineage designation.^27,28^ More than thirty amino acid mutations have been identified within Omicron spike protein, some of which are shared with other variants. In this study, we first investigated the spike diversity of the Omicron variants along with the shared spike mutations between Omicron and other variants of concern (VOCs) and variants of interest (VOIs). The Omicron spike amino acid sequences archived during the early transmission phase, and released in the GISAID database (submitted before 15 January 2022) were accessed, including 52,563 high-quality Omicron spike sequences (representing 49,609 BA.1 and 2954 BA.2 sequences). We demonstrate that the emerging and circulating Omicron subvariants originate in part through recombination with other variants. We find Revertant haplotypes in the BA.1 subvariant. Most notably, multiple characteristic amino acid mutations in the Delta spike protein have been also identified in the “Deltacron”-like Omicron Variants.

## Results

### The common mutations among Omicron (BA.1 and BA.2) and variants of concern (VOCs)

Circulating Omicron variant consists of two main subvariants, BA.1 and BA.2. BA.1 subvariant was more frequently detected than BA.2 during the early transmission phase. However, BA.2 is replacing BA.1 as the dominant epidemic subvariant in more and more countries over time.

Through scanning 52,563 high-quality completed Omicron spike gene sequences, most Omicron spike mutations appear stable (frequency >99%). 18 core mutations (frequency >99%) of BA.1 subvariant exist in NTD (A67V, del69-70, T95I, G142D, and del143-145), SD (underpinning subdomain) near the S1/S2 cleavage site (T547K, D614G, H655Y, N679K, and P681H), and S2 (D796Y, N856K, Q954H, N969K, and L981F) (Table 1). With regards to BA.2 subvariant, 27 core mutations were identified (Table 1).

**Table 1:** Comparison of Spike protein amino acid mutations between the Omicron subvariants and other VOCs and VOIs

Spike region		Position	Mutation (BA.1)	Frequency (BA.1)	Percentage	Mutation (BA.2)	Frequency (BA.2)	Percentage	Mutation in VOCs/VOIs	New mutation
		19	–	–	–	T19I	2953	100.00%	–	–
		24	–	–	–	deletion	2513	85.10%	–	–
		25	–	–	–	deletion	2513	85.10%	–	–
		26	–	–	–	deletion	2513	85.10%	–	–
		27	–	–	–	A27S	2513	85.10%	–	–
		67	A67V	49,475	99.73%	–	–	–	–	–
		69	Deletion	49,385	99.55%	–	–	–	Alpha(del69)	–
		70	Deletion	49,382	99.54%	–	–	–	Alpha(del70)	–
		95	T95I	49,513	99.81%	–	–	–	Mu(T95I)	–
	NTD	142	G142D	49,424	99.63%	G142D	2934	99.36%	–	–
		143	Deletion	49,439	99.66%	–	–	–	–	–
		144	Deletion	49,439	99.66%	–	–	–	Alpha(del144), Mu(Y144S)	–
		145	Deletion	49,440	99.66%	–	–	–	Mu(Y145N)	–
		211	Deletion	47,840	96.43%	–	–	–	–	–
		212	N212I	47,839	96.43%	–	–	–	–	–
		213	–	–	–	V213G	2950	99.90%	–	–
		214	insertE	44,368	89.44%	–	–	–	–	–
		214	insertP	44,353	89.41%	–	–	–	–	–
	–	214	insertE	44,365	89.43%	–	–	–	–	–
		339	G339D	48,729	98.23%	G339D	2953	100.00%	–	–
		346	R346K	16,819	33.90%	–	–	–	Mu(R346K)	Yes
		371	S371L	48,297	97.36%	S371F	2949	99.86%	–	–
S1		373	S373P	48,322	97.41%	S373P	2952	99.97%	–	–
		375	S375F	48,316	97.39%	S375F	2951	99.93%	–	–
		376	–	–	–	T376A	2949	99.86%	–	–
		405	–	–	–	D405N	2949	99.86%	–	–
		408	–	–	–	R408S	2946	99.76%	–	–
		417	K417N	44,711	90.13%	K417N	2952	99.97%	Beta(K417N), Gamma(K417T)	–
		440	N440K	46,470	93.67%	N440K	2926	99.09%	–	–
	RBD	446	G446S	46,892	94.52%	–	–	–	–	–
		452	L452R	897	1.81%	–	–	–	Delta(L452R)	Yes
		477	S477N	48,185	97.13%	S477N	2952	99.97%	–	–
		478	T478K	48,320	97.40%	T478K	2952	99.97%	Delta(T478K)	–
		484	E484A	48,024	96.81%	E484A	2952	99.97%	Beta/Gamma/Mu (E484K)	–
		493	Q493R	47,999	96.75%	Q493R	2953	100.00%	–	–
		496	G496S	47,965	96.69%	–	–	–	–	–
		498	Q498R	47,917	96.59%	Q498R	2953	100.00%	–	–
		501	N501Y	47,933	96.62%	N501Y	2953	100.00%	Alpha/Beta/Gamma/Mu (N501Y)	–
	–	505	Y505H	47,888	96.53%	Y505H	2952	99.97%	–	–
		547	T547K	49,496	99.77%	–	–	–	–	–
		614	D614G	49,568	99.92%	D614G	2953	100.00%	Alpha/Beta/Gamma/Delta (N501Y)	–
	SD	655	H655Y	49,509	99.80%	H655Y	2953	100.00%	Gamma(H655Y)	–
		679	N679K	49,523	99.83%	N679K	2953	100.00%	–	–
	–	681	P681H	49,515	99.81%	P681H	2953	100.00%	Alpha/Mu(P681H), Delta(P681R)	–
		701	A701V	2729	5.50%	–	–	–	Beta(A701V)	Yes
	FP	764	N764K	49,046	98.87%	N764K	2953	100.00%	–	–
		796	D796Y	49,338	99.45%	D796Y	2952	99.97%	–	–
S2	–	856	N856K	49,488	99.76%	–	–	–	–	–
		954	Q954H	49,559	99.90%	Q954H	2953	100.00%	–	–
	HR1	969	N969K	49,537	99.85%	N969K	2953	100.00%	–	–
		981	L981F	49,373	99.52%	–	–	–	–	–

*VOCs* variants of concern, *VOIs* variants of interest

52,563 high-quality Omicron spike gene sequences (49,609 BA.1 sequences, and 2954 BA.2 sequences) released before 15 January 2022 were analyzed. The mutations that have appeared in more than 800 sequences were used in this analysis

**Fig. 1:**
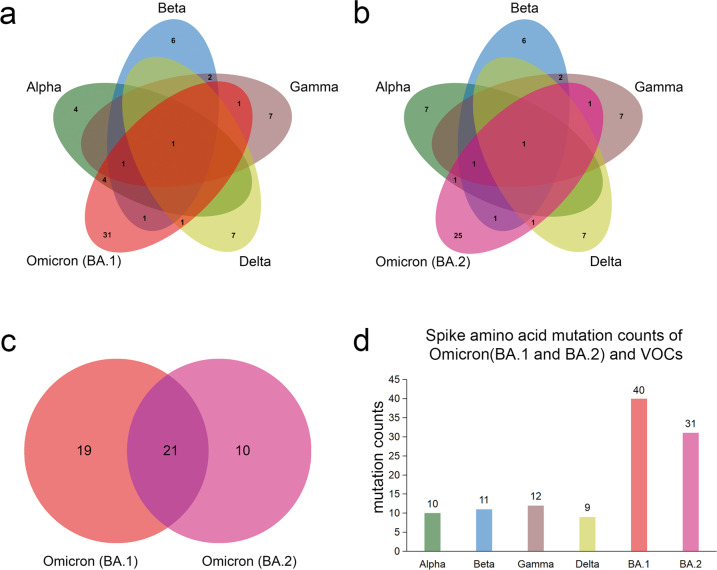
Spike protein amino acid mutations of the Omicron subvariants (BA.1 and BA.2) compared with mutations from the other four variants of concern (VOCs). **a** Venn diagram noting mutations of Omicron (BA.1) and those of VOCs. **b** Venn diagram of Omicron (BA.2) mutations compared to ones of VOCs. **c** Venn diagram of mutations between Omicron (BA.1) and Omicron (BA.2). **d** Spike protein amino acid mutation counts of Omicron subvariants (BA.1 and BA.2) compared with mutations of VOCs

BA.1 subvariant shares nine common amino acid mutations (del69-70, delY144, K417N, T478K, N501Y, D614G, H655Y, and P681H) in the spike protein with most VOCs, suggesting a possible origin of Omicron from these VOCs. Among these shared mutations, six common ones were found in Alpha variant (del69-70, delY144, N501Y, D614G, and P681H), to which the mutations of del69-70, delY144, and P681H are exclusive; three mutations were found in Beta variant (K417N, N501Y, and D614G), to which the mutation K417N is exclusive; three mutations found in Gamma (N501Y, D614G, and H655Y), to which the mutation H655Y is exclusive; two mutations found in Delta (T478K and D614G), to which the mutation T478K is exclusive (Fig. 1a and Table 1). The seven Omicron mutations exclusive to other four VOCs suggested a possible recombination origin of Omicron.

Compared to BA.1 subvariant, BA.2 shares only six amino acid mutations (K417N, T478K, N501Y, D614G, H655Y, and P681H) in the spike protein with most VOCs. Among these shared mutations, three mutations were found in Alpha variants (N501Y, D614G, and P681H); there were no del69-70 and delY144 mutations. The other three mutations in Beta, three mutations in Gamma, and two mutations in Delta were identical in the BA.2 and BA.1 genomes (Fig. 1b and Table 1).

BA.1 and BA.2 subvariants share 21 spike amino acid mutations: One in the N-terminal domain (NTD) (G142D), twelve in the receptor binding domain (RBD) (G339D, S373P, S375F, K417N, N440K, S477N, T478K, E484A, Q493R, Q498R, N501Y, and Y505H), four in SD (D614G, H655Y, N679K, and P681H), and four in S2 (N764K, D796Y, Q954H, and N969K) (Fig. 1c and Table 1).

In contrast to BA.2 subvariant, BA.1 share three additional amino acid deletions (del69-70, delY144) with the Alpha variants, suggesting a closer relationship between the BA.1 and Alpha variants (Fig. 1a, b and Table 1). Among the 21 shared mutations between BA.1 and BA.2 subvariants, 15 are specific to Omicron (G142D, G339D, S371L, S373P, S375F, N440K, S477N, Q493R, Q498R, Y505H, N679K, N764K, D796Y, Q954H, and N969K). Among these mutations, G142D in NTD, and G339D, S371L, S373P, S375F, N440K, S477N, Q493R, Q498R, and Y505H in RBD may contribute to the higher immune escape and transmissibility of Omicron variants (Table 1). As a whole, Omicron subvariants have a high number of amino acid mutations in the spike gene (40 in BA.1, and 31 in BA.2), of which some were found in other VOCs: Alpha (10x), Beta (11x), Gamma (12x), and Delta (9x). These mutations mainly occur in NTD and RBD (Fig. 1d and Table 1).

### Novel mutations and mutations with decreased frequency in the spike gene of Omicron BA.1 and BA.2

We investigated additional mutations among recently emerged BA.1 isolates and identified eight novel mutations in Omicron variant which were also found in other VOCs and VOIs (Table 2). For example, mutations R346K (33.90% of 49,609 BA.1 sequences) was found in Mu variants; A701V (5.50%) was found in Beta variants; L5F (0.37%) was found in Iota variants; and T76I (0.10%) was found in Lambda variants. Most notably, multiple representative amino acid mutations in the Delta spike protein were also identified in the recently emerged Omicron subvariants (del156-167, R158G, L452R, and P681R, at percentages of 0.14%, 0.14%, 1.81%, and 0.12%, respectively. This implied possible recombination events between the Omicron and Delta strains during the pandemic. The first “Deltacron”-like Omicron strain was isolated on November 11, 2021 in South Africa, followed on November 23 in Botswana. This indicates that the recombination between Omicron and Delta strains may occur during the early transmission phase. The other newly noted mutations (L141F, F643L, I1081V, S1147L, and P1162S) may have originated independently (Table 2).

**Table 2:** Novel mutations identified in the spike protein of the recently emerged Omicron subvariants (emerged before 15 January 2022; frequency > 50 sequences)

Spike region		Position	New Mutation	Frequency	Percentage	Mutation in VOCs & VOIs	Early event occurrence time	First country emerging
S1	–	5	L5F	184	0.37%	Iota(L5F)	2021.11.19	South Africa
	NTD	76	T76I	51	0.10%	Lambda (T76I)	2021.11.26	Netherlands
		141	L141F	56	0.11%	–	2021.11.19	South Africa
		142	G142Y	51	0.10%	–	2021.12.16	USA
		156	deletion	68	0.14%	Delta(del156)	2021.12.13	USA
		157	deletion	71	0.14%	Delta(del157)	2021.12.13	USA
		158	R158G	69	0.14%	Delta(E158G)	2021.12.15	USA
	RBD	346	R346K	16819	33.90%	Mu(R346K)	2021.11.4	Canada
		452	L452R	897	1.81%	Delta(L452R)	2021.11.11	South Africa
	SD	643	F643L	138	0.28%	–	2021.11.29	Thailand
		681	P681R	62	0.12%	Delta(P681R)	2021.11.23	Botswana
S2	–	701	A701V	2729	5.50%	Beta(A701V)	2021.11.10	South Africa
	–	1081	I1081V	351	0.71%	–	2021.11.18	U.K.
		1147	S1147L	120	0.24%	–	2021.12.13	USA
		1162	P1162S	60	0.12%	–	2021.12.10	Canada

**Fig. 2:**
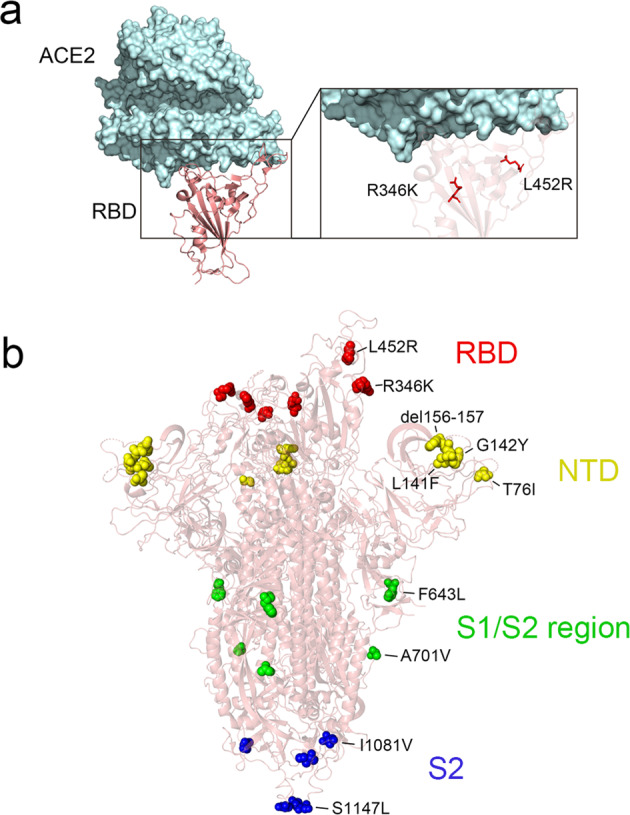
Structure of the Spike protein with amino acid mutations detected in Omicron BA.1 subvariant. **a** Structure of human ACE2 receptor complexed with SARS-CoV-2 Omicron RBD, mapped with the recent mutations. **b** Structure of SARS-CoV-2 Omicron spike protein mapped with the novel mutations. Mutated residues in each domain of the spike protein are annotated in color (red: RBD; yellow: NTD; green: S1/S2; blue: S2) using with Pymol 2.0 software through SARS-CoV-2 Omicron model PDB:7WBL and 7QO7

Several novel mutations were reported to be related to spike protein function, resulting in an enhancement of virus infectivity or in viral immune escape. Mutations that occurred in RBD, e.g., R346K, could result in a relatively weakened neutralizing antibody effect.^29^ A L452R mutation may provide evasion from cellular immunity and increased infectivity.^5^ The P681R as well as F643L and A701V mutations, near the S1/S2 cleavage site, may be associated with enhanced fusogenicity and pathogenicity of SARS-CoV-2 Delta variants.^8^ Additionally, mutations T76I, L141F, G142Y, 156–167 deletion, and R158G, located in the NTD region, were noted to affect antibody binding efficiencies and contribute to immune escape.^30^ These mutations sites are mapped and shown in Fig. 2a, b.

Apparent revertant mutations are found in some dominant mutations (frequency >95%) in the BA.1 subvariant during the pandemic. Examples are the mutations in NTD (del211 and N212I) and RBD (G339D, S371L, S373P, S375F, K417N, 440K, G446S, S477N, T478K, E484A, Q493R, G496S, Q498R, N501Y, and Y505H). The frequency of insertions of the amino acids EPE at site 214 in BA.1 decreased during the pandemic from more than 95% on 1 December 2021 to 89% on 15 January 2022. However, BA.2 spike protein remained constant (frequency >99%), with the exception of the three amino acid deletion (LPP) found at amino acids 24–26, which decreased from more than 95% frequency on 1 December 2021 to 85% on 15 January 2022 (Table 1). This may possibly be due to selection pressure on the circulating Omicron strains.

### Diverse haplotypes of Omicron spike gene and full genome phylogenetic trees show multiple recombination events during the pandemic

**Fig. 3:**
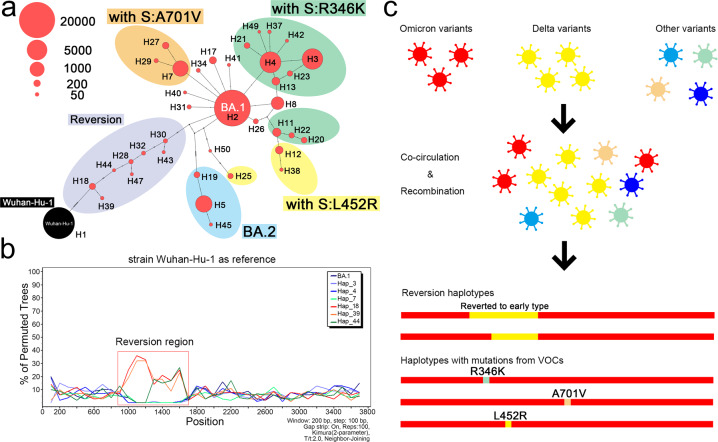
Phylogenetic network and scanning of the spike gene from representative Omicron subvariant sequences. **a** Representative Omicron spike protein haplotypes (each consisting of at least 50 sequences) were constructed with PopART using the median-joining method. Nucleotide changes were notated with lines. The spike gene from Wuhan-Hu-1 strain was set as the root. The number of sequences in each haplotype were modified into different orders of magnitude, and subgroups based on the mutation types were delineated by color. **b** BootScan analysis of revertant and representative haplotypes of Omicron spike gene. Representative spike Omicron haplotypes (H3, H4, H7) sequences and selected reversion haplotypes (H18, H39, H44) sequences are included. Bootscan map was constructed by Simplot 3.5.1 (https://sray.med.som.jhmi.edu/SCRoftware/SimPlot/) using neighboring-joining method with 100 bootstrap replicates. Wuhan-Hu-1 spike sequences were set as references and reversion region was annotated. **c** Overview of possible evolution mechanism of reversion haplotypes and haplotypes with mutations from Delta and other variants

The spike gene of Omicron subvariants consists of 49 representative haplotypes (each occurring in more than 50 sequences). BA.1, BA.2, R346K, L452R, and A701V, and a revertant type were identified in the phylogenetic network analysis (Fig. 3a). A large number of BA.1 spike mutations delineated haplotype 2, R346K, L452R, and A701V clusters and formed distinct subgroups (detailed mutations defining each haplotype are listed in Supplementary Table 1).

Multiple nucleotide mutations were detected in the haplotypes compared with BA.1, e.g., haplotype 19 and the revertant subgroup (H30, 32, and 43, etc.) and BA.2. The S:L452R subgroup (“Deltacron”-like Variants) consists of different haplotypes with multiple nucleotide substitutions, indicating a possibly separate origin of S:L452R haplotypes or prior recombination events. Haplotype 25 in S:L452R subgroup, with multiple nucleotide differences compared with BA.1, could have resulted through recombination between Omicron and Delta variants, gaining the mutation S:L452R from Delta and losing multiple mutations from Omicron (Fig. 3a). Some of these “Deltacron” -like haplotypes are being tracked by the UK Health Security Agency (https://www.gov.uk/government/publications/sars-cov-2-variants-of-public-health-interest/sars-cov-2-variants-of-public-health-interest-25-february-2022) and underway to confirm by Santé publique France (https://t.co/tVAKmHRYSy). The revertant subgroup consisted of Omicron haplotypes in which several BA.1 representative mutations were lost and appeared to have reverted to the bases of the Wuhan-Hu-1 strain. Multiple nucleotide differences in other haplotypes occurred, likely as multiple independent mutation events, or perhaps as recombination events among highly similar sequences. Bootscan analysis of Omicron spike sequences also indicated that the reversion haplotypes (H18, H39, and H44) were more similar to Delta variants when compared to typical Omicron haplotypes (Fig. 3b).

Furthermore, single nucleotide differences could also originate from recombination events among highly similar strains. Loops detected in phylogenetic networks also indicate possible recombination events among highly similar Omicron variants or subvariants (Fig. 3a, c). Multiple newly detected or recent mutations in the Omicron spike gene make it possible to trace a putative mutation origin from representative mutations in VOIs or VOCs, especially the Delta variant, which suggests possible recombination events between Omicron and Delta variants (Table 2).

**Fig. 4:**
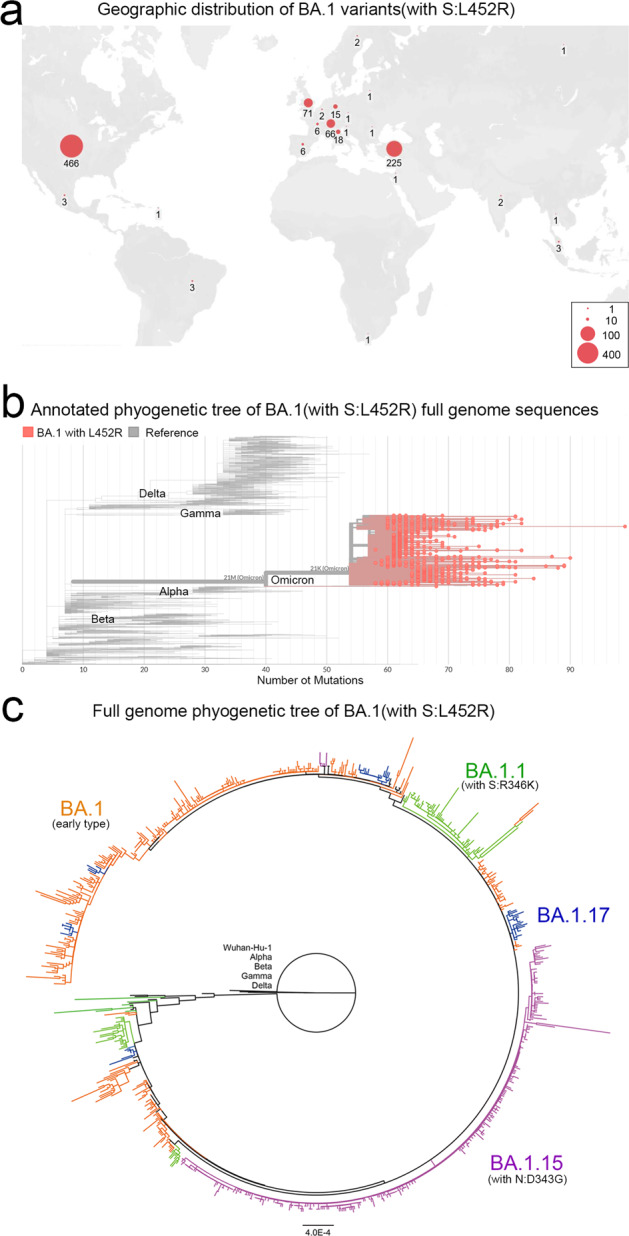
Geographic distribution and whole genome analyses of “Delatcron”-like BA.1 subvariants (with S:L452R mutations). **a** Geographic distribution of “Delatcron”-like BA.1 subvariants (with S:L452R), with the number of genome sequences noted. **b** Whole genome annotation of amino acid mutations highlighting the “Deltacron”-like BA.1 subvariants (with S:L452R). 897 full genome sequences of BA.1 with S:L452R mutation were shown with red circles. **c** Phylogenetic analysis of the “Deltacron”-like BA.1 subvariants (with S:L452R). Maximum likelihood tree was constructed using Iqtree2 with 1000 bootstrap replicates and TIM + F + R3 model. Low quality sequences were excluded. 897 SARS-CoV-2 BA.1 full genome sequences with S:L452R mutation submitted to the GISAID database before 15 January 2022 and reference sequences from VOCs were included. Pango sublineages BA.1, BA.1.1, BA.1.15, and BA.1.17 are marked in colors

For further investigating the geographic distribution and genome diversity of the “Deltacron”-like variants, 897 Omicron genomic sequences of high quality containing S:L452R mutation reported for the Delta variant were analyzed (Fig. 4a). “Deltacron”-like variants were mainly distributed in North America, Europe, and West Asia (Fig. 4a). Whole genome annotation of amino acid mutations and phylogenetic tree corroborated the diversity among these S:L452R containing “Deltacron”-like Omicron genomes, which consist Omicron Pango sublineages BA.1, BA.1.1 (with S:R346K), BA.1.15, and BA.1.17 (Fig. 4b, c). BA.1 and BA.1.15 are the two major sublineages that acquired S:L452R mutation. The mutation profiles among whole genomes of BA.1 are diverse, and the sequences branched to diverse clades by phylogenetic analyses.

## Discussion

Virus co-infection and recombination can amplify pathogenicity, for example, the well-studied “model organism” adenovirus,^14–20^ and also in coronaviruses.^21,22,24^ SARS-CoV-2 has been shown to co-infect and recombine.^31,32^ In host populations with disproportionate immunocompromised conditions, such as in Africa,^33^ the possibility of long-term infections of SARS-CoV-2 variants may be higher than in populations otherwise healthy and/or vaccinated. For example, on 10 June 2021, a passenger on a flight from Johannesburg, South Africa to Shenzhen, China tested positive for SARS-CoV-2. The patient was found to be coinfected with two SARS-CoV-2 variants: Beta and Delta, with the ratio of the relative abundance between the two variants maintained at 1:9 (Beta: Delta) in a 14-day period.^31^ Furthermore, putative evidence of recombination in the Orf1ab and spike genes was shown.^28^ Such recombination events may not be rare, especially considering that there are hundreds of variants circulating in the general population. In USA, during November 2021 and February 2022, Helix sequenced 29,719 positive SARS-COV-2 samples and identified 20 co-infections.^34^ In French, Delta/Omicron SARS-CoV-2 co-infections were identified in seven immunocompetent and epidemiologically unrelated patients during the fifth wave of COVID-19 pandemics.^32^ These co-infections were detected by PCR assays targeting SARS-CoV-2 S:K417N and S:L452R and confirmed by whole genome sequencing. Another case report described prolonged infectious SARS-CoV-2 shedding up to 70 days from an asymptomatic immunocompromised individual with cancer.^35^ A SARS-CoV-2 isolated from her presented with four new mutations within the spike protein and also eight in structural proteins and polymerase region. The marked within-host genomic evolution of SARS-CoV-2 with continuous turnover of dominant viral variants was observed. Under reduced immune pressure or immune-suppression, long-term infections create conditions and increase the likelihood of simultaneous co-infections with multiple SARS-CoV-2 variants, and optimizing conditions for genome recombination.

Co-infections of different SARS-CoV-2 variants in the population will accelerate their evolution through recombination. Among the Omicron subvariants and VOCs, many shared mutations were identified in this study. We speculate that some of the Omicron spike protein mutations resulted from co-infections of variants. Recombination among diverse variants may have contributed to the shared presence of different mutations between the VOCs. For example, the BA.1 subvariant has three more Alpha-related mutations (del69-70, delY144) than BA.2, and therefore may be phylogenetically closer to the Alpha variant, suggesting that Alpha or other unknown variants that carry these mutations may have contributed to the emergence of the BA.1 subvariant (Table 1). Multiple mutation differences causing reversion haplotypes may have originated from the recombination between the Omicron and other variants (Fig. 3 and Supplementary Table 1).

Except the shared mutations, many other mutations (30 in BA.1 and 25 in BA.2) could not be accounted for among previous dominant variants (Fig. 1). Mass novel spike mutations emerged in Omicron variants at the same time are quite unusual. Previous study suggested the animal origin of Omicron variants and possible zoonotic transmission events due to the Omicron mutation types.^36^ The zoonotic transmission events from deer to human and minks to human were identified, indicating that the risk of zoonotic transmission actually exists.^37^ The Spike mutation Q493R and Q498R were reported in infected mice, but rare in human.^38^ The selection pressure in animals may cause mass novel mutations in early Omicron variants, which are subsequently selected in human under selection pressure. This is confirmed by our finding that the frequency of some original BA.1 mutations has decreased during the circulation (Table 1). As previous research reported, Omicron variants seem to have more waning clinic outcomes and lower replication capacity in vitro.^39^ The unusual superiority of Omicron variants to compete the Delta variants may be due to its immune escape ability. It was previously reported that evolution of SARS-CoV-2 in an immunosuppressed COVID-19 patient led to immune escape variants.^40,41^ Deletions in NTD, for example, delY144, were detected in multiple immunosuppressed COVID-19 patients, which were associated with the immune escape. Selection pressure from population with immunity against SARS-CoV-2 may play an important role in virus evolution. The initial recombination events among variants may occur randomly in immunocompromised population, but are selected by subsequent pressure from herd immunity. The ability of immune escape from pre-existing antibodies may drive the variant evolution.

A recently reported recombinant SARS-CoV-2 “Deltacron”, and its genome sequences, elicited controversy and concerns of sequencing errors and sample contamination. Nevertheless, it was confirmed that co-infections by Omicron and Delta variants have already occurred in specific populations (https://www.gov.uk/government/publications/sars-cov-2-variants-of-public-health-interest/sars-cov-2-variants-of-public-health-interest-11-february-2022). Recombination among the extant variants may lead to the emergence of new variants. A total of 10 cases of “Deltacron” are underway to confirm by Santé publique France (SPF) (https://t.co/tVAKmHRYSy). Recently, two independent cases of infection by a Delta/Omicron recombinant virus were identified in USA.^34^ In our study, multiple VOC and VOI mutations were detected in Omicron variants circulating before 15 January 2022 (Fig. 1 and Table 1). The integration of these mutations may lead to changes in phenotype. Five additional typical amino acid mutations in Delta variants were also identified in recently emergent Omicron isolates (before 15 January 2022) (Table 2).

Although the frequency of “Deltacron”-like sequences (with S:L452R mutation) increases during the pandemic, from 1937 on 15 January 2022 when the data were collected, to 6727 on 24 March 2022, the percentage of “Deltacron”-like sequences remains still low (lower than 1%). The S:L452R mutation may increase the adaption ability of “Deltacron”-like variants compared to the original Omicron variants due to its increasing immune escape. However, the competition of the virus transmission may depend on many factors. For example, the Omicron BA.2 subvariant occurred later and remains low frequency before 15 January 2022, but it now exceeds BA.1 subvariant in many continents in March 2022 (Supplementary Fig. 1). Omicron BA.2 subvariants carry 27 core mutations in spike compared to 18 core mutations for BA.1 (Table 1 and Supplementary Fig. 2). Single mutation integration of Omicron BA.1 subvariants may be not enough to compete the BA.2 subvariant with more mutations. Another reason is that the vaccine-induced or preexisting immunity promotes the variants with stronger immune evasion dominant. In general, the wide-reaching infection is associated with not only the virus infectivity but the immune escape ability. Interestingly, we also detected more than 100 BA.2 sequences with S:L452R mutation. The influence of these recombined variants remains further monitoring.

S:R346K mutation, previously reported in Mu variants, is also identified in Omicron BA.1 variants (named as Omicron BA.1.1) (Tables 1, 2) with higher frequency than the early BA.1 variant (Supplementary Fig. 1). This mutation is related to virus immune escape and may increase the viral adaption.^29^ S:A701V mutation related to *Beta* variants was also identified in Omicron variants. It may increase the cleavage efficiency of furin.^8^

Beyond the spike gene focused in this study, we further detected possible recombination events in other genes among Omicron and Delta variants. For example, typical nucleocapsid protein (N) mutations from Delta variants were also detected within nine “Deltacron”-like sequences (with S:L452R mutation), when compared to the early Omicron strains (Supplementary Fig. 2). N protein is associated with the virus pathogenesis and immunity response.^42,43^ Whether these mutations change the virus pathogenicity or immunity response still needs further investigation. Multiple recombination regions found in single strain suggest active diverse recombination events among the Omicron variants.

In summary, by analyzing sequences from a large number of Omicron subvariants, we identified diverse recombination events between two Omicron subvariants and several SARS-CoV-2 VOCs/VOIs, including “Deltacron”-like variants, suggesting that co-infection and subsequent genome recombination play important roles in the on-going evolution of SARS-CoV-2. Some of the recombination events may have led to modifications in protein function and viral fitness. Continued monitoring of SARS-CoV-2 genomes for mutations is critically important to our understanding of its evolution and impact on human health, and is also essential for the recognition of changes to viral epitopes that would require vaccine modifications. Prevention and control of the spread of SARS-CoV-2 in immunocompromised and unvaccinated populations may contribute to slowing the generation of recombinant SARS-COV-2 variants.

## Materials and methods

### Omicron sequence dataset

958,062 full-length SARS-CoV-2 Omicron spike sequences and Omicron genomic sequences of high quality containing S:L452R mutation collected before January 15,2022, were downloaded from the GISAID EpiCoV^TM^ Database (http://www.GISAID.org). 53,056 complete high quality Omicron spike sequences were filtered for downstream analysis. The spike sequences with gaps or degenerate bases were excluded.

### Sequences alignment and mutation analysis

Sequences were aligned by MAFFT version 7 online serve with default parameter (https://mafft.cbrc.jp/alignmeloadnt/server/) and BioEdit, using Wuhan-Hu-1 (NC_045512) strain as reference.^44,45^ Spike amino mutations were analyzed and calculated with BioAider V1.334 using Wuhan-Hu-1 (NC_045512) strain and Omicron haplotypes as reference.^3,46^ D structures were constructed using Pymol 2.0 software through SARS-CoV-2 Omicron model PDB:7WBL and 7QO7.^47–49^ Venn diagrams were created using jvenn.^50^

### Haplotype phylogenetic network and phylogenetic tree analyses

Omicron sequence haplotypes and frequency were calculated using R package tidyfst. Haplotypes with more than 5 sequences were output. Haplotypes format were output through with DnaSP 6.0,^51^ and a subsequent phylogenetic network was constructed using PopART software (http://popart.otago.ac.nz/index.shtml) by median-join method with epsilon 0. Mutation and Pango lineage of “Deltacron”-like variants with L452R mutation was annotated with Nextclade (https://clades.nextstrain.org).^52^ Phylogenetic tree analyses were conducted with Iqtree2, applying the maximum-likelihood method with 1000 bootstrap replicates using TIM + F + R3 model selected by MFP ModelFinder.^53^ Reference sequences were download from NCBI and GISAID database, including Alpha (OL689430.1), Beta (EPI_ISL_1040757), Gamma (EPI_ISL_1445071), and Delta (OK091006.1).The trees were annotated and modified using Figtree (version 1.4.4) (https://github.com/rambaut/figtree/releases). Omicron sublineage distribution was analyzed through COVID CG website APP.^54^

### Sequence recombination analysis

The spike sequences in Reversion haplotypes were analyzed compared with dominant Omicron spike haplotypes (Frequency > 1000). The spike sequences of strain Wuhan-Hu-1 was set as reference.^55^ Bootscan analysis was performed using Simplot software (version 3.5.1) (https://sray.med.som.jhmi.edu/SCRoftware/SimPlot/), Kimura (2-parameter), neighboring-joining method, with window 200 bp and step 100 bp.

## Supplementary information

Supplementary_Materials

## Data Availability

All data are available in the manuscript, the supplementary materials, or from GISAID’s EpiCoV™ Database (www.gisaid.org).
