# Supplementary material for: Tracking SARS-CoV-2 Omicron diverse spike gene mutations identifies multiple inter-variant recombination events
Source: Signal Transduct Target Ther. 2022 Apr 26;7:138. doi: 10.1038/s41392-022-00992-2 (PMC9039610; doi:10.1038/s41392-022-00992-2)
Supplement: Supplementary file 1 — Supplementary_Materials [file 41392_2022_992_MOESM1_ESM.pdf]

Supplementary Materials for

Tracking SARS-CoV-2 Omicron diverse spike gene mutations identifies multiple inter-variant recombination events

Junxian Ou[1,2], Wendong Lan[2,] Xiaowei Wu[2], Tie Zhao[1], Biyan Duan[1], Peipei Yang[1], Yi Ren[1], Lulu Quan[2], Wei Zhao[2], Donald Seto[3], James Chodosh[4], Zhen Luo[1,5] Jianguo Wu[1,5*] & Qiwei Zhang[1,2,5*]

[1]Guangdong Provincial Key Laboratory of Virology, Institute of Medical Microbiology, Jinan University, Guangzhou 510632, China

[2]BSL-3 Laboratory (Guangdong), Guangdong Provincial Key Laboratory of Tropical Disease Research, School of Public Health, Southern Medical University, Guangzhou 510515, China

[3]Bioinformatics and Computational Biology Program, School of Systems Biology, George Mason University, Manassas, VA 20110, USA

[4]Department of Ophthalmology, Howe Laboratory Massachusetts Eye and Ear, Harvard Medical School, Boston, MA 02114, USA

[5]Foshan Institute of Medical Microbiology, Foshan 528315, China

Correspondence to: zhangqw@jnu.edu.cn (QZ), jwu898@jnu.edu.cn (JW)

This PDF file includes:

Supplementary Fig. 1
Supplementary Fig. 2
Supplementary Table 1

Supplementary Fig. 1

Timecourse of Omicron variant sublineage distribution (https://covidcg.org/?tab=home).

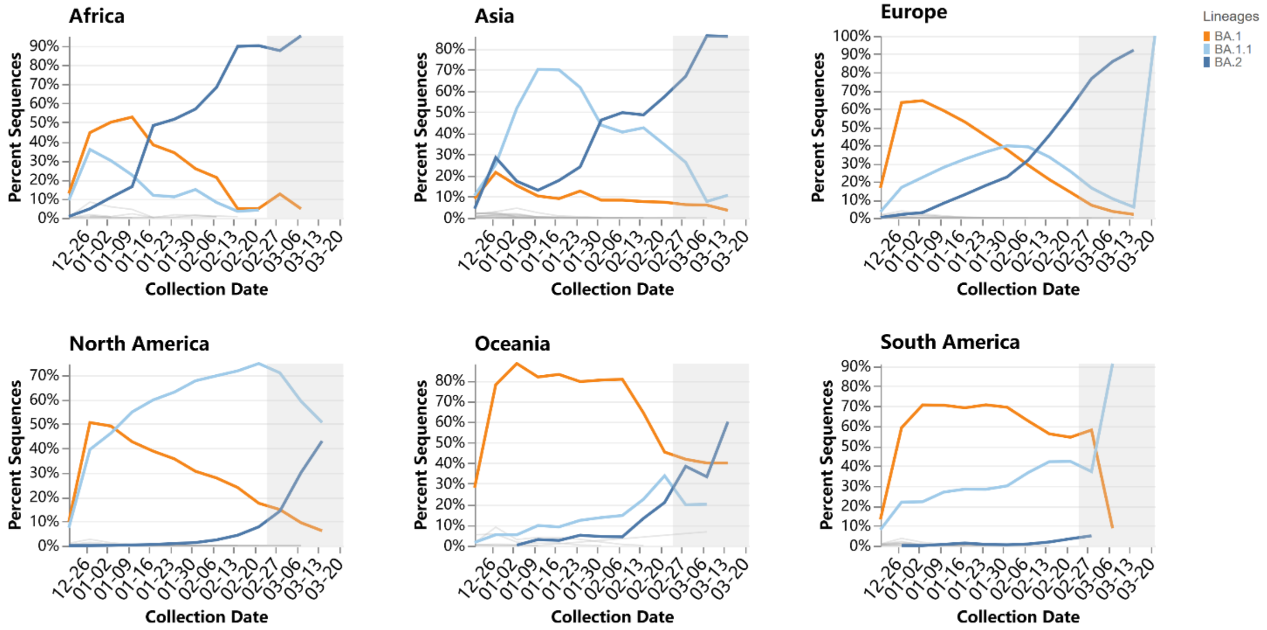

Supplementary Fig. 2

The possible recombinations from Delta variants were detected within N gene of "Deltacron" -like Omicron variants (with S:L452R mutation) when compared to the early Omicron strains.

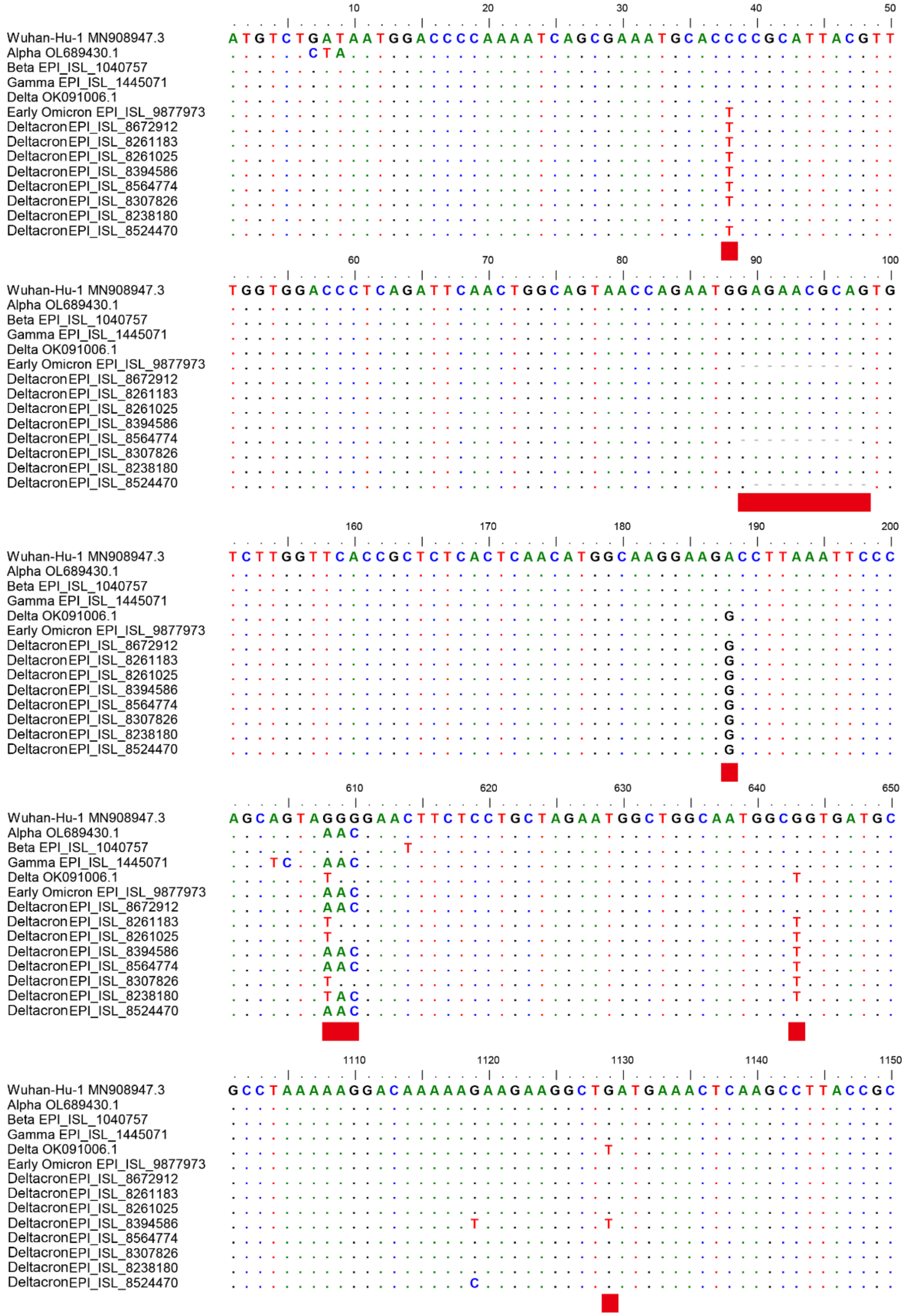

Supplementary Table 1. The annotation of haplotypes of Omicron spike protein.

Haplotype	aa Substitutions	Insertions	aa Deletions	Counts
1	Reference	Reference	Reference	Reference
2	A67V,T95I,Y145D,L212I,G339D,S371L,S373P,S375F,K417N,N440K,G446S,S477N,T478K,E484A,Q493R,G496S,Q498R,N501	214EPE	H69-,V70-,G142-,V143-,Y144-,N211-	19745
3	A67V,T95I,Y145D,L212I,G339D,R346K,S371L,S373P,S375F,K417N,N440K,G446S,S477N,T478K,E484A,Q493R,G496S,Q498	214EPE	H69-,V70-,G142-,V143-,Y144-,N211-	6406
4	A67V,T95I,Y145D,L212I,G339D,R346K,S371L,S373P,S375F,K417N,N440K,G446S,S477N,T478K,E484A,Q493R,G496S,Q498	214EPE	H69-,V70-,G142-,V143-,Y144-,N211-	6093
5	T19I,A27S,G142D,V213G,G339D,S371F,S373P,S375F,T376A,D405N,R408S,K417N,N440K,S477N,T478K,E484A,Q493R,Q498R,N501Y,Y505H,D6		L24-,P25-,P26-	2061
6	A67V,T95I,Y145D,L212I,G339D,S371L,S373P,S375F,K417N,N440K,G446S,S477N,T478K,E484A,Q493R,G496S,Q498R,N501Y,Y505H,T547K,D6		H69-,V70-,G142-,V143-,Y144-,N211-	1942
7	A67V,T95I,Y145D,L212I,G339D,S371L,S373P,S375F,K417N,N440K,G446S,S477N,T478K,E484A,Q493R,G496S,Q498R,N501	214EPE	H69-,V70-,G142-,V143-,Y144-,N211-	1586
8	A67V,T95I,Y145D,L212I,G339D,S371L,S373P,S375F,N440K,G446S,S477N,T478K,E484A,Q493R,G496S,Q498R,N501Y,Y505	214EPE	H69-,V70-,G142-,V143-,Y144-,N211-	573
9	A67V,T95I,Y145D,G339D,S371L,S373P,S375F,K417N,N440K,G446S,S477N,T478K,E484A,Q493R,G496S,Q498R,N501Y,Y505H,T547K,D614G,H6		H69-,V70-,G142-,V143-,Y144-	414
10	T19I,G142D,V213G,G339D,S371F,S373P,S375F,T376A,D405N,R408S,K417N,N440K,S477N,T478K,E484A,Q493R,Q498R,N501Y,Y505H,D614G,H655Y,N679K,P681H,N764K,D796Y,Q954H,N			397
11	A67V,T95I,Y145D,L212I,G339D,S371L,S373P,S375F,S477N,T478K,E484A,Q493R,G496S,Q498R,N501Y,Y505H,T547K,D614	214EPE	H69-,V70-,G142-,V143-,Y144-,N211-	365
12	A67V,T95I,Y145D,L212I,G339D,S371L,S373P,S375F,L452R,S477N,T478K,E484A,Q493R,G496S,Q498R,N501Y,Y505H,T547	214EPE	H69-,V70-,G142-,V143-,Y144-,N211-	306
13	A67V,T95I,Y145D,L212I,G339D,R346K,S371L,S373P,S375F,N440K,G446S,S477N,T478K,E484A,Q493R,G496S,Q498R,N501	214EPE	H69-,V70-,G142-,V143-,Y144-,N211-	267
14	A67V,T95I,Y145D,L212I,G339D,S371L,S373P,S375F,N440K,G446S,S477N,T478K,E484A,Q493R,G496S,Q498R,N501Y,Y505H,T547K,D614G,H6		H69-,V70-,G142-,V143-,Y144-,N211-	261
15	A67V,T95I,Y145D,L212I,G339D,R346K,S371L,S373P,S375F,K417N,N440K,G446S,S477N,T478K,E484A,Q493R,G496S,Q498R,N501Y,Y505H,T5		H69-,V70-,G142-,V143-,Y144-,N211-	237
16	A67V,T95I,Y145D,L212I,G339D,S371L,S373P,S375F,K417N,N440K,G446S,S477N,T478K,E484A,Q493R,G496S,Q498R,N501Y,Y505H,T547K,D6		H69-,V70-,G142-,V143-,Y144-,N211-	233
17	A67V,T95I,Y145D,L212I,G339D,S371L,S373P,S375F,K417N,N440K,G446S,S477N,T478K,E484A,Q493R,G496S,Q498R,N501	214EPE	H69-,V70-,G142-,V143-,Y144-,N211-	208
18	A67V,T95I,Y145D,T547K,D614G,H655Y,N679K,P681H,N764K,D796Y,N856K,Q954H,N969K,L981F		H69-,V70-,G142-,V143-,Y144-	206
19	T19I,A27S,G142D,V213G,G339D,S371F,S373P,S375F,T376A,D405N,R408S,K417N,N440K,S477N,T478K,E484A,Q493R,Q498R,N501Y,Y505H,D6		L24-,P25-,P26-	187
20	A67V,T95I,Y145D,L212I,G339D,R346K,S371L,S373P,S375F,S477N,T478K,E484A,Q493R,G496S,Q498R,N501Y,Y505H,T547	214EPE	H69-,V70-,G142-,V143-,Y144-,N211-	184
21	A67V,T95I,Y145D,L212I,G339D,R346K,S371L,S373P,S375F,K417N,N440K,G446S,S477N,T478K,E484A,Q493R,G496S,Q498	214EPE	H69-,V70-,G142-,V143-,Y144-,N211-	178
22	A67V,T95I,Y145D,L212I,G339D,R346K,S371L,S373P,S375F,S477N,T478K,E484A,Q493R,G496S,Q498R,N501Y,Y505H,T547	214EPE	H69-,V70-,G142-,V143-,Y144-,N211-	164
23	A67V,T95I,Y145D,L212I,G339D,R346K,S371L,S373P,S375F,N440K,G446S,S477N,T478K,E484A,Q493R,G496S,Q498R,N501	214EPE	H69-,V70-,G142-,V143-,Y144-,N211-	159
24	A67V,T95I,Y145D,L212I,G339D,R346K,S371L,S373P,S375F,K417N,N440K,G446S,S477N,T478K,E484A,Q493R,G496S,Q498R,N501Y,Y505H,T5		H69-,V70-,G142-,V143-,Y144-,N211-	158
25	A67V,T95I,Y145D,L452R,S477N,T478K,E484A,Q493R,G496S,Q498R,N501Y,Y505H,T547K,D614G,H655Y,N679K,P681H,N764K,D796Y,N856K,Q9		H69-,V70-,G142-,V143-,Y144-	149
26	A67V,T95I,Y145D,L212I,G339D,S371L,S373P,S375F,K417N,G446S,S477N,T478K,E484A,Q493R,G496S,Q498R,N501Y,Y505	214EPE	H69-,V70-,G142-,V143-,Y144-,N211-	140
27	A67V,T95I,Y145D,L212I,G339D,S371L,S373P,S375F,K417N,N440K,G446S,S477N,T478K,E484A,Q493R,G496S,Q498R,N501	214EPE	H69-,V70-,G142-,V143-,Y144-,N211-	138
28	A67V,T95I,Y145D,L212I,G339D,S371L,S373P,S375F,N440K,G446S,T547K,D614G,H655Y,N679K,P681H,N764K,D796Y,N856	214EPE	H69-,V70-,G142-,V143-,Y144-,N211-	110
29	A67V,T95I,Y145D,L212I,G339D,S371L,S373P,S375F,K417N,N440K,G446S,S477N,T478K,E484A,Q493R,G496S,Q498R,N501	214EPE	H69-,V70-,G142-,V143-,Y144-,N211-	99
30	A67V,T95I,Y145D,L212I,G339D,S371L,S373P,S375F,K417N,N440K,G446S,S477N,T478K,E484A,T547K,D614G,H655Y,N679	214EPE	H69-,V70-,G142-,V143-,Y144-,N211-	98
31	A67V,T95I,Y145D,L212I,G339D,S371L,S373P,S375F,K417N,N440K,G446S,S477N,T478K,E484A,Q493R,G496S,Q498R,N501	214EPE	H69-,V70-,G142-,V143-,Y144-,N211-	95
32	A67V,T95I,Y145D,L212I,G339D,S371L,S373P,S375F,K417N,N440K,G446S,T547K,D614G,H655Y,N679K,P681H,N764K,D796	214EPE	H69-,V70-,G142-,V143-,Y144-,N211-	93

33	A67V, T95I, Y145D, G339D, S371L, S373P, S375F, K417N, N440K, G446S, S477N, T478K, E484A, Q493R, G496S, Q498R, N501Y, Y505H, T547K, D614G, H6		H69-, V70-, G142-, V143-, Y144-	91
34	A67V, T95I, Y145D, L212I, G339D, S371L, S373P, S375F, K417N, N440K, G446S, S477N, T478K, E484A, Q493R, G496S, Q498R, N501	214EPE	H69-, V70-, G142-, V143-, Y144-, N211-	86
35	A67V, T95I, Y145D, G339D, S371L, S373P, S375F, K417N, N440K, G446S, S477N, T478K, E484A, Q493R, G496S, Q498R, N501Y, Y505H, T547K, D614G, H6		G142-, V143-, Y144-	75
36	A67V, T95I, Y145D, G339D, S371L, S373P, S375F, K417N, N440K, G446S, S477N, T478K, E484A, Q493R, G496S, Q498R, N501Y, Y505	214EPE	H69-, V70-, G142-, V143-, Y144-	70
37	A67V, T95I, Y145D, L212I, G339D, R346K, S371L, S373P, S375F, K417N, N440K, G446S, S477N, T478K, E484A, Q493R, G496S, Q498	214EPE	H69-, V70-, G142-, V143-, Y144-, N211-	66
38	A67V, T95I, Y145D, L212I, G339D, S371L, S373P, S375F, L452R, S477N, T478K, E484A, Q493R, G496S, Q498R, N501Y, Y505H, T547	214EPE	H69-, V70-, G142-, V143-, Y144-, N211-	65
39	A67V, T95I, Y145D, T547K, D614G, H655Y, N679K, P681H, N764K, D796Y, N856K, Q954H, N969K, L981F		H69-, V70-, G142-, V143-, Y144-	65
40	L5F, A67V, T95I, Y145D, L212I, G339D, S371L, S373P, S375F, K417N, N440K, G446S, S477N, T478K, E484A, Q493R, G496S, Q498R,	214EPE	H69-, V70-, G142-, V143-, Y144-, N211-	63
41	A67V, T95I, Y145D, L212I, G339D, S371L, S373P, S375F, K417N, N440K, G446S, S477N, T478K, E484A, Q493R, G496S, Q498R, N501	214EPE	H69-, V70-, G142-, V143-, Y144-, N211-	63
42	A67V, T95I, Y145D, L212I, G339D, R346K, S371L, S373P, S375F, K417N, N440K, G446S, S477N, T478K, E484A, Q493R, G496S, Q498	214EPE	H69-, V70-, G142-, V143-, Y144-, N211-	62
43	A67V, T95I, Y145D, L212I, G339D, R346K, S371L, S373P, S375F, K417N, N440K, G446S, S477N, T478K, E484A, T547K, D614G, H655	214EPE	H69-, V70-, G142-, V143-, Y144-, N211-	62
44	A67V, T95I, Y145D, L212I, G339D, S371L, S373P, S375F, T547K, D614G, H655Y, N679K, P681H, N764K, D796Y, N856K, Q954H, N969	214EPE	H69-, V70-, G142-, V143-, Y144-, N211-	61
45	T19I, A27S, G142D, V213G, G339D, S371F, S373P, S375F, T376A, D405N, R408S, K417N, N440K, S477N, T478K, E484A, Q493R, Q498R, N501Y, Y505H, D6		L24-, P25-, P26-	58
46	A67V, T95I, Y145D, L212I, G339D, S371L, S373P, S375F, K417N, N440K, G446S, S477N, T478K, E484A, Q493R, G496S, Q498R, N501Y, Y505H, T547K, D6		H69-, V70-, G142-, V143-, Y144-, N211-	58
47	A67V, T95I, Y145D, L212I, G339D, R346K, S371L, S373P, S375F, N440K, G446S, T547K, D614G, H655Y, N679K, P681H, N764K, D796	214EPE	H69-, V70-, G142-, V143-, Y144-, N211-	55
48	A67V, T95I, Y145D, L212I, G339D, R346K, S371L, S373P, S375F, N440K, G446S, S477N, T478K, E484A, Q493R, G496S, Q498R, N501Y, Y505H, T547K, D6		H69-, V70-, G142-, V143-, Y144-, N211-	55
49	A67V, T95I, Y145D, L212I, G339D, R346K, S371L, S373P, S375F, K417N, N440K, G446S, S477N, T478K, E484A, Q493R, G496S, Q498	214EPE	H69-, V70-, G142-, V143-, Y144-, N211-	54
50	A67V, T95I, Y145D, L212I, K417N, N440K, G446S, S477N, T478K, E484A, Q493R, G496S, Q498R, N501Y, Y505H, T547K, D614G, H655	214EPE	H69-, V70-, G142-, V143-, Y144-, N211-	52
